# Emissionsarme Bauprodukte im Innenraum: Zur Arbeit des Ausschusses zur gesundheitlichen Bewertung von Bauprodukten (AgBB)

**DOI:** 10.1007/s00103-026-04247-1

**Published:** 2026-05-26

**Authors:** Ana Maria Scutaru, Ole Johanns

**Affiliations:** 1 Geschäftsstelle Ausschuss zur gesundheitlichen Bewertung von Bauprodukten (AgBB), Fachgebiet II 1.3 „Innenraumhygiene und gesundheitsbezogene Umweltbelastungen“, Umweltbundesamt, Corrensplatz 1, 14195 Berlin, Deutschland; 2 Vorsitzender Ausschuss zur gesundheitlichen Bewertung von Bauprodukten (AgBB), Referat Umweltbezogener Gesundheitsschutz, Behörde für Justiz und Verbraucherschutz Hamburg, Billstraße 80a, 20539 Freie und Hansestadt Hamburg, Deutschland

Innenräume von Gebäuden sind zentrale Lebens- und Arbeitsräume für uns Menschen. Tatsächlich verbringen wir rund 80–90 % unseres Tages in Innenräumen. Bauprodukte können durch die Freisetzung unterschiedlicher flüchtiger organischer Verbindungen (VOC) eine für die Gesundheit bedeutende nachteilige Schadstoffquelle der Innenraumluft darstellen. In den 1990er-Jahren führte das wachsende Bewusstsein für potenzielle Quellen von VOC in Innenräumen zu umfangreichen Forschungsaktivitäten zu den Emissionscharakteristika relevanter Produkte und zur Entwicklung geeigneter Prüfnormen. Europäische Wissenschaftlerinnen und Wissenschaftler arbeiteten an diesen Themen im Rahmen des Projekts „European Collaborative Action“ (ECA), das von der Gemeinsamen Forschungsstelle (Joint Research Centre) in Ispra (Italien) geleitetet wurde. Der 1997 veröffentlichten ECA-Forschungsbericht, Bericht Nr. 18 „Bewertung von VOC-Emissionen aus Bauprodukten – feste Bodenbeläge“, hatte maßgeblichen Einfluss auf die Entwicklung von Verfahren zur Bewertung von Bauproduktemissionen in mehreren europäischen Ländern.

In Deutschland wurde im Jahr 1997 der „Ausschuss zur gesundheitlichen Bewertung von Bauprodukten“ (AgBB) von den zuständigen Behörden aus den Bereichen Umwelt und Gesundheit des Bundes und der Länder gemeinsam mit den Baubehörden gegründet. Der AgBB nutzte den ECA-Bericht Nr. 18 als Grundlage für die Definition von Kriterien für die gesundheitsbezogene Bewertung von in Aufenthaltsräumen zu verwendenden Bauprodukten, wie sie von den Landesbauordnungen gefordert werden.

In dieser Ausgabe des Bundesgesundheitsblattes finden Sie erstmals eine amtliche Mitteilung des AgBB zur gesundheitlichen Bewertung der Emissionen von VOC aus Bauprodukten – das AgBB-Bewertungsschema 2026. Die erste Fassung dieses Bewertungsschemas wurde im Jahr 2000 auf der Homepage des Deutschen Instituts für Bautechnik (DIBt) veröffentlicht. Damals diente der Bewertungsmaßstab als Vorschlag für eine einheitliche und reproduzierbare gesundheitsbezogene Bewertung von Bauprodukten und sollte eine Diskussion mit relevanten Akteuren, insbesondere unter den Bauproduktherstellern, anstoßen. Bis heute bilden 3 zentrale Prinzipien die Grundlage des AgBB-Bewertungsschemas: 1) Bewertung von Stoffen mit bekannten toxikologischen Eigenschaften anhand von NIK-Werten (NIK: niedrigste interessierende Konzentration), 2) Schwellenwerte für nicht bewertbare oder unbekannte Stoffe sowie 3) Schwellenwerte für die Gesamtmenge chemischer Emissionen.

Das Verfahren des AgBB sah ursprünglich einen 2‑stufigen Bewertungsmaßstab vor. Dabei stellte die „Eignung für die Verwendung in Innenräumen“ eine verbindliche Mindestanforderung für innenraumrelevante Produkte dar. Darüber hinaus war eine höhere Qualitätsstufe für Produkte mit besonders geringen Emissionen vorgesehen. Die Entwicklung des Bewertungsschemas wurde nach seiner ersten Veröffentlichung in einem intensiven fachlichen Austausch mit Verbänden, Herstellern, Prüfstellen und weiteren Fachleuten begleitet. Im Ergebnis wurde der Fokus auf die Stufe „Eignung für die Verwendung in Innenräumen“ als nachvollziehbare und transparente Mindestanforderung gelegt. Nach einer Einführungsphase und auf Grundlage umfangreicher Emissionsdaten erwies sich das AgBB-Bewertungsschema als grundsätzlich geeignet und belastbar.

Die höheren Qualitätsanforderungen des AgBB-Bewertungsschemas für freiwillige Gütesiegelsysteme (veröffentlicht 2001) wurden für die Zertifizierung von Produkten mit besonders geringen Emissionen vorgeschlagen. Bei freiwilliger Anwendung bleiben die Kernkriterien unverändert; lediglich Summenparameter können strengere Vorgaben enthalten. Diese strengeren AgBB-Anforderungen bilden seit 2002 die Grundlage für die emissionsbezogenen Aspekte des Gütezeichens Blauer Engel. Das AgBB-Bewertungsschema wird hier nicht nur für Bauprodukte, sondern auch für Möbel und Spielzeug angewendet.

Das AgBB-Bewertungsschema ist seit 2004 im deutschen Baurecht verankert. Auch international hat es Einfluss genommen. So sind die AgBB-Kriterien sowie der Verweis auf die deutsche NIK-Liste seit 2014 im belgischen Dekret zur Begrenzung von VOC-Emissionen aus Bodenbelägen enthalten. Auch das dänische Gütezeichen Danish Indoor Climate Labelling übernahm im Jahr 2018 die Kriterien des AgBB-Bewertungsschemas, einschließlich der NIK-Liste. Beide Beispiele verdeutlichen, dass die AgBB-Strategie zur Bewertung von Bauproduktemissionen zu einem breit anwendbaren und genutzten Maßstab und zu einer Art europäischem Standard geworden ist.

Das AgBB-Bewertungsschema wird kontinuierlich weiterentwickelt und fortgeschrieben. Grundlage dafür sind aktuelle wissenschaftliche Erkenntnisse, insbesondere aus durch das Umweltbundesamt vergebenen Forschungsprojekten, die im fachlichen Austausch mit allen beteiligten Akteuren diskutiert und berücksichtigt werden. Diese kontinuierliche Weiterentwicklung ist auch deshalb besonders relevant, weil die zunehmende Luftdichtheit moderner Gebäude den Bedarf an emissionsarmen Materialien für Innenräume in den letzten Jahren weiter verstärkt hat. Das AgBB-Verfahren galt anfänglich bei einigen Akteuren als zu komplex und zu teuer, hat jedoch seine Effektivität, Eignung und Praktikabilität unter Beweis gestellt. Im Laufe der Jahre hat es sich als hoher Standard für die gesundheitsbezogene Bewertung von Produkten etabliert.

Das AgBB-Bewertungsschema 2026 im amtlichen Teil dieser Ausgabe enthält die Vorgehensweise der Bauproduktprüfung, die fachlichen Grundlagen sowie die stofflichen Bewertungen anhand der aktuellen NIK-Liste.

Zusammenfassend halten wir fest: Das AgBB-Bewertungsschema schafft in Bezug auf den Gesundheitsschutz gegenüber den Emissionen von VOC aus Bauprodukten in seiner Anwendung und Umsetzung Transparenz und Vergleichbarkeit. Hersteller wissen dadurch, welche Anforderungen sie erfüllen müssen. Prüfstellen verfügen über normierte Prüfverfahren. Und Bauherren, Planer und Architekten können sich hinsichtlich der fachgerechten Produktauswahl darauf verlassen, dass nach dem AgBB-Bewertungsschema geprüfte Bauprodukte nach einheitlichen Kriterien und Maßstäben bewertet wurden.

Wir wünschen Ihnen viel Freude bei der Lektüre dieser Ausgabe des *Bundesgesundheitsblattes*!

## Supplementary Information

English_version_editorial_issue_6_2026

## Data Availability

Es handelt sich um ein Editorial zur Entstehung des AgBB und zur Arbeit des AgBB. Die Informationen in diesem Artikel sind auch im eingereichten AgBB Manuskript enthalten. Die Informationen und Daten zu diesem Artikel wurden vom AgBB erstellt.

