# Supplementary material for: Emissionsarme Bauprodukte im Innenraum: Zur Arbeit des Ausschusses zur gesundheitlichen Bewertung von Bauprodukten (AgBB)
Source: Bundesgesundheitsblatt Gesundheitsforschung Gesundheitsschutz. 2026 May 26;69(6):621–2. [Article in German] doi: 10.1007/s00103-026-04247-1 (PMC13212384; doi:10.1007/s00103-026-04247-1)
Supplement: Supplementary file 1 — English_version_editorial_issue_6_2026 [file 103_2026_4247_MOESM1_ESM.pdf]

Editorial

This is an English translation of the German editorial: Emissionsarme Bauprodukte im Innenraum: Zur Arbeit des Ausschusses zur gesundheitlichen Bewertung von Bauprodukten (AgBB) (Bundesgesundheitsblatt (06/2026)). The responsibility for the translation lies solely with the authors. Please note that only the original German-language editorial can be cited.

Low-emission building products for indoor use: On the work of the Committee for the Health-related Evaluation of Building Products (AgBB)

Ana Maria Scutaru[1], Ole Johanns[2]

[1] Office of the Committee for the Health-related Evaluation of Building Products (AgBB), Section II 1.3 ‘Indoor hygiene, health-related environmental impacts’, German Environment Agency, Berlin, Germany
[2] Chair of the Committee for the Health-related Evaluation of Building Products (AgBB), Ministry of Justice and Consumer Protection, Free and Hanseatic City of Hamburg, Germany

The interiors of buildings are central living and working spaces. In fact, we spend around 80 to 90% of our day indoors. By releasing various volatile organic compounds (VOCs), building products can represent a significant source of indoor air pollution that is harmful to health. In the 1990s, growing awareness of potential sources of VOCs indoors led to extensive research into the emission characteristics of relevant products and the development of suitable testing standards. European scientists worked on these issues as part of the ‘European Collaborative Action’ (ECA) project led by the Joint Research Centre (JRC) in Ispra (Italy). One of the ECA research reports published in 1997, Report No. 18 ‘Evaluation of VOC emissions from building products – solid flooring materials’, had a significant influence on the development of methods for assessing emissions from building products in several European countries.

In Germany, the Committee for the Health Assessment of Construction Products (AgBB) was established in 1997 by the federal and state authorities responsible for the area of environment and health, in collaboration with the building authorities. The AgBB used the ECA Report No. 18 as basis for defining criteria for the health-related assessment of building products intended for use in interior spaces, as required by the state building regulations.

In this issue of the Bundesgesundheitsblatt, you will find, for the first time, an official communication from the AgBB on the health evaluation of VOC emissions from building products – the AgBB evaluation scheme 2026. The first version of the AgBB evaluation scheme was published on the website of the Deutsche Institut für Bautechnik (DIBt) in 2000. At the time, the assessment criteria served as proposal for a uniform and reproducible health-related evaluation of building products and was intended to stimulate discussion among relevant stakeholders, particularly among building products manufacturers. To this day, the AgBB evaluation scheme is based on three core principles: 1) the assessment of substances with known toxicological properties using LCI values (LCI: lowest concentration of interest); 2) threshold values for substances that cannot be assessed or for which no data is available; 3) threshold values for the total quantity of chemical emissions.

The AgBB procedure described originally two quality levels. The quality level, 'suitability for indoor use', constituted a mandatory minimum requirement for products intended for indoor use. In addition, a higher quality level was envisaged for products with particularly low emissions. Following its first publication, the development of the evaluation scheme was accompanied by an intensive technical dialogue with products associations, manufacturers, testing bodies and other experts. As a result, the focus was placed on the 'suitability for indoor use' quality level as a clear and transparent minimum requirement. Following an introductory phase and based on extensive emissions data, the AgBB evaluation scheme proved to be fundamentally suitable and robust.

The higher quality requirements of the AgBB evaluation scheme for voluntary labelling schemes (published in 2001) were proposed for the certification of products with particularly low emissions. Under voluntary application, the core criteria remain unchanged; only sum parameters may be stricter. Since 2002, these stricter AgBB requirements have formed the basis for the emissions-related aspects of the Blue Angel label. The AgBB evaluation scheme is applied here not only to construction products, but also to furniture and toys.

The AgBB evaluation scheme has been embedded in German building law since 2004. It has also had an impact internationally. The AgBB criteria, as well as the reference to the German LCI list, have been incorporated in 2014 into the Belgian decree on the limitation of VOC emissions from floor coverings. The Danish Indoor Climate Labelling scheme also adopted in 2018 the criteria of the AgBB evaluation scheme, including the LCI list. Both examples show that the AgBB strategy for assessing emissions from building products has become a widely applicable and used benchmark and a kind of European standard.

The AgBB evaluation scheme is continuously developed and updated. This is based on current scientific findings, mainly from projects commissioned by the German Environment Agency, which are discussed and taken into account through technical consultation with all stakeholders involved. This ongoing development is particularly relevant because the increasing airtightness of modern buildings has further heightened the demand for low-emission materials for interior spaces in recent years. Although the AgBB procedure was initially regarded by some stakeholders as too complex and too expensive, it has since proven its effectiveness, suitability and practicality. Over the years, it has established itself as a high standard for the health-related assessment of products.

The AgBB evaluation scheme 2026 presented in the official section of this issue includes the procedure for testing building products, the technical principles and the substance assessments based on the current LCI list. For the English version of the AgBB evaluation scheme 2026, please visit the corresponding website.

To sum up: the AgBB evaluation scheme ensures transparency and comparability in its application and implementation with regard to health protection against VOC emissions from building products. This means that manufacturers know what requirements they need to meet. Testing bodies have standardised test procedures. And building owners, planners and architects can be confident in their product selection, that building products tested in accordance with the AgBB evaluation scheme have been assessed against uniform criteria and standards.

Conflict of interest
Ana Maria Scutaru and Ole Johanns declare no conflict of interest.

Corresponding address

Dr. Ana Maria Scutaru
Office of the Committee for the Health-related Evaluation of Building Products (AgBB),
Section II 1.3 ‘Indoor hygiene, health-related environmental impacts’,
German Environment Agency,
Corrensplatz 1, 14195 Berlin, Germany
E-Mail: anamaria.scutaru@uba.de

Dr. Ole Johanns

Chair, AgBB – Committee for Health-related Evaluation of Building Products,

Free and Hanseatic City of Hamburg,

Authority for Justice and Consumer Protection,

Unit for Environmental Health Protection,

Billstraße 80a, 20539 Hamburg, Germany

E-Mail: ole.johanns@justiz.hamburg.de
